# Comparison of Different Anthropometric Measurements and Inflammatory Biomarkers

**DOI:** 10.1155/2012/124693

**Published:** 2012-05-17

**Authors:** Yaron Arbel, Edo Y. Birati, Itzhak Shapira, Talya Finn, Shlomo Berliner, Ori Rogowski

**Affiliations:** Departments of Cardiology and Internal Medicine “D” and “E”, Tel Aviv Sourasky Medical Center, Sackler Faculty of Medicine, Tel Aviv University, Tel Aviv 62439, Israel

## Abstract

*Introduction.* Different anthropometric variables have been shown to be related to cardiovascular morbidity and mortality. Our aim was to compare the association between different anthropometric measurements and inflammatory status. *Methods and results.* A cross-sectional study design in which we analyzed the data collected during a five-year period in the Tel Aviv Medical Center Inflammation Survey (TAMCIS). Included in the study were 13,033 apparently healthy individuals at a mean (SD) age of 43. Of these, 8,292 were male and 4,741 female. A significant age-adjusted and multiple-adjusted partial correlation was noted between all anthropometric measurements and all inflammatory biomarkers. There was no significant difference in the correlation coefficients between different biomarkers and anthropometric variables. *Conclusion.* Most of the common used anthropometric variables are similarly correlated with inflammatory variables. The clinician can choose the variable that he/she finds easiest to use.

## 1. Introduction

Different anthropometric variables have been shown to be related to cardiovascular morbidity and mortality. Of these variables, body mass index (BMI) and waist circumference are amongst the most common and part of the metabolic syndrome. In addition, it has been shown that peripheral fat deposition is associated with less severe, and central obesity related to more severe, cardiovascular disease [1–3].

It is well known that inflammation and central obesity are related [4, 5], however, there is a lack of agreement regarding which anthropometric variables have the best correlation with inflammation. Since atherosclerosis is an inflammatory disease [6], we set out to compare the correlation between different anthropometric measurements and inflammation. Such knowledge will help in defining which physical measure should be used in evaluating proinflammatory body habitus. 

## 2. Methods and Procedures

### 2.1. Participants

We have presently analyzed data that has been collected during the last five years in the Tel Aviv Medical Center Inflammation Survey (TAMCIS), a registered data bank of the Israeli Ministry of Justice [7]. This is a relatively large cohort of individuals who attended our medical centre for a routine annual checkup and gave their written informed consent for participation according to the instructions of the institutional ethics committee. A total of 17,393 subjects gave their informed consent (10,975 males, 6,418 females). Later, 3,030 subjects were excluded from the analysis due to any malignancy, immunosuppressive therapy, known inflammatory diseases (arthritis, inflammatory bowel disease, psoriasis, etc.), pregnancy, steroidal or nonsteroidal treatment (except for aspirin at a dose of ≤325 mg/day), acute infection, or invasive procedures (surgery, catheterization, etc.) during the prior 6 months. We further excluded 535 individuals with a history of a proven atherothrombotic event (myocardial infarction, cerebrovascular event or peripheral arterial occlusive disease) and 556 due to diabetes mellitus. Finally, an additional 239 subjects were excluded due to missing data relating to any of their anthropometric measurements or the inflammatory variables. Following these exclusions the study group comprised 13,033 individuals (8,292 men and 4,741 women). 

### 2.2. Definition of Risk Factors

Results of the routine health checkup were assessed employing certain definitions in order to identify atherothrombotic risk factors in individuals. These included diabetes mellitus which was defined as a fasting blood glucose concentration of ≥7.0 mmol/L (126 mg/dL) or the intake of insulin or oral hypoglycemic medications. Hypertension was defined as a blood pressure of ≥140/90 mmHg on two separate measurements or the use of antihypertensive medications. Dyslipidemia was defined according to the low density lipoprotein (LDL) or nonhigh density lipoprotein (non-HDL) cholesterol concentrations for those individuals displaying elevated triglyceride concentrations of >2.26 mmol/L (200 mg/dL) above the recommended levels (according to the risk profile defined by the updated ATP III recommendations [8]) or as using lipid lowering medications. Smokers were defined as individuals who smoked at least 5 cigarettes per day while past smokers had to have stopped smoking for at least 30 days prior to examination. 

### 2.3. Anthropometric Measurements

In this study, we used common anthropometric measurements including: waist (cm), weight (kg), BMI (kg/m^2^), BAI (body adiposity index), waist to hip ratio, and waist to height ratio. Of these variables, BAI is a new index that is less known. It is defined as ((hip circumference)/((height)^1.5^) − 18) that is better associated with percent of body fat [9]. 

### 2.4. Laboratory Methods

The white blood cell count (WBCC) and differential were performed by using the Coulter STKS (Beckman Coulter, Nyon, Switzerland) electronic cell analyzer, quantitative fibrinogen by the method of Clauss [10], and a Sysmex 6000 (Sysmex-Corporation, Hyaga, Japan) autoanalyzer while the high sensitivity C-reactive protein (hs-CRP) was performed by using a Behring BN II Nephelometer (DADE Behring, Marburg, Germany).

### 2.5. Statistical Analysis

All data was summarized and displayed as mean (standard deviation (SD)) for the continuous variables and as number of patients (expressed as a percentage) in each group for the categorical variables. Since the hs-CRP and triglyceride concentrations displayed irregular distributions, we used logarithmic transformation which converted the distributions to normal ones for all statistical procedures. Therefore all results of hs-CRP and triglyceride concentrations are expressed as back transformed geometrical means. The one-way Kolmogorov-Smirnov test was used to assess the distributions. 

Pearson's partial correlations for confounding variables were performed to evaluate the association between the different anthropometric measurements and the different inflammatory variables. All correlations were adjusted for age. 

In order to evaluate and compare the different anthropometric measurements and their contribution to the variability of the different inflammatory variables we performed linear regression models, with the inflammatory variables as the dependent variables and many potential and known parameters as the independent variables. The parameters entered into the model in addition to the different anthropometric measurements were age, complete lipid profile including LDL, HDL, and triglycerides, diastolic and systolic blood pressure measurements, glucose concentration, alcohol consumption, sport intensity, number of schooling years (as a measure of socioeconomic status), medications including aspirin, beta blockers, calcium channel blockers, angiotensin converting enzyme (ACE) inhibitors, angiotensin II receptor blockers (ARBs), statins, fibrates, and cardiovascular risk factors including current and past smoking status and family history of coronary heart disease (CHD). For women, the models included the addition of two other variables—oral contraceptive or hormonal replacement therapy usage. The level of significance used for all of the above analyses was two tailed, *P* < 0.05. The SPSS statistical package was used to perform all statistical evaluation (SSPS Inc., Chicago, IL, USA).

## 3. Results

We have presently analyzed a total of 13,033 individuals (8,292 male and 4,741 female) at a mean (SD) age of 43 (11) years. The characteristic age, blood pressure, lipid profile, alcohol consumption, sport intensity, as well as the five anthropometric measurements, are presented in Table 1, while the respective percentage of individuals with different cardiovascular risk factors and relevant medications are presented in Table 2. Since women have higher inflammatory variables and different anthropometric dimensions, we present the data according to gender. As expected, men had a higher weight and waist dimension (*P* < 0.001).  Table 3 presents the different inflammatory variables in the cohort. Women had higher hsCRP and fibrinogen values (*P* < 0.001). A significant age-adjusted Pearson partial correlation was noted between all anthropometric measurements and the concentration of hs-CRP, fibrinogen and total white blood cell count in both genders and is presented in Table 4. There were higher correlation coefficients in the female gender group. 

In order to evaluate and compare the different anthropometric variables as potential contributors to the variability of the different inflammatory variables we performed linear regression models with various inflammatory variables and cardiovascular risk factors, medications, and anthropometric measurements as potential confounders, and the results of the partial correlation of the anthropometric variables are presented in Table 5. As can be seen, even after controlling for many confounders, there was a strong correlation between anthropometric measures and inflammatory biomarkers. The commonly used BMI and waist circumference, but also the less known waist to height ratio, were the anthropometric variables which demonstrated the strongest association with the inflammatory biomarkers. The correlation was stronger in women.

## 4. Discussion

There are multiple lines of evidence to suggest an association between low grade inflammation, dysmetabolism, and vascular disease. In this regard, it has been convincingly shown that anthropometric measures are important for clinical and epidemiological assessments. It might be relevant to determine what the relations are, if any, between them and the presence of low grade inflammation. Such a relation might support the link between abnormal measures and the presence of abnormal metabolism.

The main finding of our study is that among the commonly used inflammatory biomarkers that are available in daily practice (hs-CRP, quantitative fibrinogen, and WBC) the hs-CRP correlates best with the different anthropometric measures. An additional relevant finding is the similar magnitude of correlation between different anthropometric variables and the different inflammatory variables with strong association for BMI and waist circumference. The correlation was stronger in females. This point is of special relevance due to the introduction of modern scales, into most of the modern clinics. They automatically calculate the individual's BMI due to their ability to measure height using optic measures.

We have shown that anthropometric measurements, particularly waist circumference and BMI, are strongly associated with inflammation. The high correlations between these variables and the different inflammatory variables were significant also after controlling for many known and possible confounding parameters. This link has clinical implementations since anthropometric measurements are used in order to evaluate the metabolic status of patients. It is well known that the visceral fat of the abdomen is proinflammatory. In fact, the fat can be infiltrated by macrophages that can lead to insulin resistance and endothelial dysfunction [2, 4, 11].

According to our results, there is no need to measure the more cumbersome hip, waist/hip, or waist/height ratios as they are no superior to simpler measurements. 

Our data is also supported by studies that show that the metabolic syndrome is closely related to the inflammatory status of the patient [12]. Since the abdominal fat is known to be proinflammatory and a main cause of the metabolic syndrome, it is also the main cause of waist circumference and BMI.

In conclusion, most of the common used anthropometric variables are similarly correlated with inflammatory variables. The clinician should choose the variable that he/she finds easiest to use.

## Figures and Tables

**Table:** (a)

Men (*N* = 8,292)	Mean	SD	25th percentile	Median	75th percentile
Age (years)	43	11	35	43	51
Waist (cm)	94	10	88	93	100
Weight (kg)	82	13	74	81	89
Waist/hip ratio	0.97	0.10	0.91	0.97	1.03
Waist/height ratio	0.54	0.06	0.49	0.53	0.57
BAI	24	4	21	23	26
BMI (kg/m^2^)	27	4	24	26	29
Systolic BP (mmHg)	124	14	115	120	130
Diastolic BP (mmHg)	78	8	71	78	82
Glucose (mg/dL)	92	9	86	91	97
HDL cholesterol (mg/dL)	50	10	43	49	56
LDL cholesterol (mg/dL)	122	31	100	120	142
Triglycerides (mg/dL)	109	2	76	108	153
Alcohol consumption (glass/week)	1.2	2.3	0	0	2
Sport intensity (hours/week)	2.3	2.7	0	2	3.3
Schooling years (years)	15	3	14	16	17

**Table:** (b)

Women (*N* = 4,741)	Mean	SD	25th percentile	Median	75th percentile
Age (years)	44	11	36	45	52
Waist (cm)	81	11	73	80	88
Weight (kg)	66	12	58	64	73
Waist/hip ratio	0.84	0.10	0.77	0.84	0.90
Waist/height ratio	0.50	0.07	0.45	0.49	0.54
BAI	29	5	25	28	31
BMI (kg/m^2^)	25	5	22	24	27
Systolic BP (mmHg)	116	15	105	114	125
Diastolic BP (mmHg)	74	8	70	72	80
Glucose (mg/dL)	88	9	82	88	94
HDL cholesterol (mg/dL)	65	15	54	63	73
LDL cholesterol (mg/dL)	117	32	94	114	136
Triglycerides (mg/dL)	89	2	63	86	121
Alcohol consumption (glass/week)	0.5	1.4	0	0	0
Sport intensity (hours/week)	1.8	2.5	0	1	3
Schooling years (years)	15	3	12	15	17

BAI: body adiposity index; BMI: Body mass index; HDL: high density lipoprotein;

LDL—low density lipoprotein.

**Table 2:** Frequency of cardiovascular risk factors and use of prescription medication in men and women.

	Men (*N* = 8,292)		Women (*N* = 4,741)	
	N	%	N	%
Current smoker	1363	16.4	856	18.1
Past smoker	2015	24.3	913	19.3
Hypertension	1853	22.3	642	13.5
Dyslipidemia	2269	27.4	884	18.6
Family history of CHD	1162	14.0	801	16.9

Aspirin	368	4.4	92	1.9
Beta blockers	252	3.0	153	3.2
Calcium channel blockers	151	1.8	55	1.2
ACE inhibitors	233	2.8	76	1.6
ARBs	53	0.6	18	0.4
Statins	617	7.4	284	6.1
Fibrates	80	1.0	23	0.5
Oral contraceptives			684	14.4
Hormonal replacement therapy			381	8.0

CHD: Coronary heart disease; ACE: Angiotensin converting enzyme; ARB: Angiotensin II receptor blocker.

**Table 3:** Mean, SD and the quartiles of the different inflammatory variables, in men (upper part) and women (lower part).

Men (*N* = 8,292)	Mean	SD	25th percentile	Median	75th percentile
hsCRP (mg/L)	1.4	2.8	0.7	1.3	2.8
Fibrinogen (mg/dL)	281	57	242	276	315
WBC (∗10^3^ cell/*μ*L)	6.8	1.6	5.6	6.6	7.6

Women (*N* = 4,741)	Mean	S.D.	25th percentile	Median	75th percentile

hsCRP (mg/L)	1.6	3.2	0.7	1.5	3.8
Fibrinogen (mg/dL)	310	59	270	307	345
WBC (∗10^3^ cell/*μ*L)	6.8	1.7	5.6	6.6	7.7

hsCRP: high-sensitivity C-reactive protein; WBC: white blood cell count.

**Table 4:** Age-adjusted Pearson partial correlation between the different anthropometric measurements and the different inflammatory variables*.

Men	Waist	Weight	BMI	BAI	Waist/Hip	Waist/Height
hsCRP	0.350	0.296	0.356	0.214	0.214	0.370
Fibrinogen	0.191	0.143	0.175	0.153	0.085	0.204
WBC	0.187	0.162	0.196	0.095	0.140	0.200

Women	Waist	Weight	BMI	BAI	Waist/Hip	Waist/Height

hsCRP	0.405	0.411	0.466	0.337	0.221	0.421
Fibrinogen	0.247	0.236	0.270	0.251	0.096	0.259
WBC	0.166	0.163	0.182	0.105	0.120	0.170

*All above correlations are highly significant (all *P* < 0.001).

**Table 5:** Partial correlation between the different anthropometric measurements and the different inflammatory variables in the linear regression models* after adjustment for many possible risk factors and medications^‡^.

Men	Waist	Weight	BMI	BAI	Waist/Hip	Waist/Height
hsCRP	0.279	0.233	0.286	0.178	0.144	0.297
Fibrinogen	0.152	0.108	0.137	0.132	0.048	0.165
WBC	0.104	0.090	0.116	0.067	0.063	0.115

Women	Waist	Weight	BMI	BAI	Waist/Hip	Waist/Height

hsCRP	0.332	0.353	0.401	0.284	0.138	0.344
Fibrinogen	0.214	0.204	0.233	0.216	0.058	0.222
WBC	0.081	0.094	0.104	0.056	0.045	0.082

*Included in the models in addition to the anthropometric measurements were age, systolic and diastolic blood pressure measurements, glucose concentration, lipid profile including HDL, LDL, and triglyceride concentration, sport intensity and alcohol consumption, number of schooling years (as a measure of socioeconomic status), cardiovascular risk factors including smoking status, family history of CHD and medications including aspirin, *beta* blockers, calcium channel blockers, ACE inhibitors, ARBs, statins, fibrates, and for women also oral contraceptives or hormonal replacement therapy.

^‡^All above correlations are highly significant (all *P* < 0.001).
